# Pharmacogenomics and genetic ancestry: an opportunity to transform clinical practice in Colombia

**DOI:** 10.3389/fphar.2026.1774562

**Published:** 2026-02-17

**Authors:** Andy A. Acosta-Monterrosa, Kevin Fernando Montoya-Quintero, Ernesto Barceló-Martinez, Johana Galván-Barrios

**Affiliations:** 1 Center for Meta-Research and Scientometrics in Biomedical Sciences, Barranquilla, Colombia; 2 Facultad de Ciencias para la Salud, Universidad de Manizales, Manizales, Colombia; 3 Clínica Colsanitas S. A., Clínica Iberoamérica, Barranquilla, Colombia; 4 Biomedical Scientometrics and Evidence-Based Research Unit, Universidad de la Costa, Barranquilla, Colombia

**Keywords:** Colombia, pedigree, pharmacogenetics, precision medicine, therapeutics

## Abstract

Pharmacogenomics has emerged as a clinically actionable discipline capable of reducing inter-individual variability in drug response, adverse drug reactions, and therapeutic failure. However, its real-world implementation remains constrained by a structural limitation: the predominance of Eurocentric genomic evidence and its limited clinical portability to genetically heterogeneous and underrepresented populations. This gap undermines the coherence between evidence generation, synthesis, and clinical application, particularly in admixed settings. Colombia represents a high-stakes case for pharmacogenomic implementation due to its marked inter-individual and regional genetic ancestry heterogeneity, coupled with a substantial and partly preventable burden of adverse drug reactions in an ageing and increasingly polypharmacy-exposed population. Direct extrapolation of pharmacogenomics panels and guidelines derived from other populations is therefore clinically risky and scientifically incoherent. In this Perspective, we propose an ancestry-based pharmacological diagnosis (APD) as a clinically oriented framework that integrates individual- and population-level genetic ancestry with actionable pharmacogenomics variants to inform drug selection, dosing, and risk stratification. APD is not a race-based or deterministic approach; rather, it treats ancestry as a probabilistic modifier of pharmacokinetic and pharmacodynamic variability, enhancing the external validity and clinical portability of pharmacogenomic guidance. We highlight CÓDIGO, a nationally led genomic consortium, as a proof-of-concept infrastructure enabling APD by providing ancestry-resolved allele frequencies and actionable variant data across Colombian populations. Finally, we discuss how APD can improve therapeutic efficacy, patient safety, health-system efficiency, equity, and scientific coherence, positioning ancestry-informed pharmacogenomics as a scalable and ethically grounded public-health strategy for precision medicine in Colombia and similar settings.

## 1 Pharmacogenomics: definition, scope, and clinical readiness

Pharmacogenomics, or pharmacogenetics (PGx) is defined as the study of how an individual’s genetic makeup influences their response to a drug, affecting both efficacy and safety. More recent definitions broaden this concept to include the use of genomic technologies to discover and develop new therapeutics, as well as to optimize drug selection and dosing for each patient, with the explicit aim of maximizing efficacy while minimizing toxicity (Pirmohamed, 2023; Aneesh et al., 2009).

This emerging field is increasingly being positioned as a new medicine with substantial potential for real-world implementation, driven by converging factors. First, inter-individual variability in drug response (particularly therapeutic failure and adverse drug reactions) is common and carries considerable clinical and economic costs. This challenge is expected to intensify with population ageing and the rising prevalence of multimorbidity and polypharmacy, thereby adding to the hospital burden (Osanlou et al., 2022). Second, genotyping technologies are becoming more accessible and less costly, making the integration of pharmacogenomics into routine care feasible as one of the earliest pathways for incorporating genomic medicine. Third, the field is no longer primarily academic: there is growing interest from public policy and health systems given its potential to improve clinical outcomes and implement safer care pathways, as well as from the pharmaceutical sector, since therapeutics supported by genetic evidence tend to have a higher likelihood of success in clinical development (Pirmohamed, 2023; King et al., 2019).

## 2 Why ancestry matters in population pharmacogenomics

Pharmacogenomics leverages specific genomic features, such as germline variants that modulate pharmacokinetics and pharmacodynamics, with downstream effects on dosing, efficacy, and adverse drug reactions (Pirmohamed, 2023). Within this framework, genetic ancestry is a central population-level determinant because the architecture and distribution of variants relevant to drug response are not homogeneous across groups. Notably, Yang et al. (Yang et al., 2021) show that ancestry-informative markers (AIMs) are enriched in pharmacogenetic loci, underscoring that trans-ancestral differentiation is consequential and should be explicitly incorporated into population pharmacogenomics studies and strategies to avoid biased extrapolations and to improve the clinical portability of findings.

## 3 The portability problem: eurocentric evidence and its clinical consequences

Despite longstanding recognition of these issues, available empirical evidence remains markedly skewed toward populations of European ancestry. In genome-wide association studies (GWAS), for example, it was reported that in 2009 approximately 96% of participants were of European ancestry; although representation improved over time, a 2016 update still showed a strong concentration in European samples (∼81%), while individuals of African, Latin American/Hispanic, and Indigenous ancestry remained <4% of the total analyzed (Popejoy and Fullerton, 2016; Petrovski and Goldstein, 2016). This European-centric bias is not merely a representativeness problem: it can reduce the replicability and transferability of variant-drug response associations to non-European populations and, in turn, bias pharmacogenomics guidelines, algorithms, and drug labels. Moreover, the overrepresentation of European ancestry in reference datasets constrains clinical interpretation for non-European individuals, who tend to yield a larger number of candidate variants, reflecting greater uncertainty and analytic burden, due to the lack of appropriate comparators, thereby reinforcing inequities in precision medicine (Petrovski and Goldstein, 2016).

## 4 Colombia as a high-stakes case for implementation

Colombia is a paradigmatic example of admixture-driven genetic heterogeneity, both across individuals and across regions, which makes it risky to directly extrapolate pharmacogenomics panels, allele frequencies, or algorithms derived from other populations. For instance, Colombian genomes from Medellín show a tri-hybrid admixture (European, Native American, and African) with a very wide inter-individual range of ancestry proportions, from near-completely European profiles to much more balanced tri-ancestral contributions, with mean estimates of ∼74.6% European, ∼18.1% Native American, and ∼7.3% African (Rishishwar et al., 2015). At a broader scale, pronounced regional gradients in ancestry distribution are evident nationwide: ancestry maps indicate higher African ancestry proportion in coastal regions (particularly the Pacific), predominance of European ancestry proportion in central areas, and relatively higher Native American ancestry proportion in the southwest and in the eastern/Amazonian regions (Ruiz-Linares et al., 2014).

Against this backdrop of marked ancestral heterogeneity, and consistent with the global driver of inter-individual variability in drug response, adverse drug reactions (ADRs) impose a substantial clinical and economic burden in the Colombian health system. In an internal medicine service, the direct cost attributable to ADR care over a 5-month period was estimated at COP $93,633,422 - $122,155,406 (USD $24,615.87 - $32,114.19; adjusted as of 14 December 2025), highlighting the contribution of medication-related expenses and prolonged length of stay (Tribiño et al., 2006). At the population level, 5,342 ADR reports linked to 468 medicines have been described, with frequently implicated drugs including metamizole, enalapril, and warfarin, indicating that this is a cross-cutting issue rather than one confined to a single service or institution (Machado-Alba et al., 2016). At the most severe end of the spectrum, Rojas-Velandia et al. reported that ADRs accounted for 13.8% of ICU admissions and that a substantial proportion was preventable or avoidable, pointing to a concrete opportunity for clinical improvement through preventive strategies and risk stratification (Rojas-Velandia et al., 2017).

Among older adults, ADRs occur within a setting of increasing vulnerability driven by ageing and clinical complexity. The proportion of the population aged >60 years rose from 9% (2005) to 13.4% (2018), a shift accompanied by higher levels of frailty and comorbidity that predispose to adverse hospital outcomes; moreover, polypharmacy (defined as ≥5 medications) is common in geriatric care and increases the risk of drug-drug interactions and ADRs (Ojeda Méndez et al., 2020). Along the same lines, a high prevalence of potentially inappropriate prescribing (PIP) has been documented (31.52%), which heightens the risk of adverse events and pharmacological interactions and is associated with greater healthcare utilization (visits, hospitalizations, readmissions, and prolonged stays), reinforcing that the ADR burden not only exists but is concentrated and amplified in older adults (Arroyave et al., 2025).

## 5 Ancestry-based pharmacological diagnosis to transform clinical practice in Colombia

Taken together, Colombia’s pronounced ancestry heterogeneity, coupled with a substantial and partly preventable burden of ADRs in an ageing and increasingly polypharmacy-exposed population, creates an urgent clinical and public-health imperative to implement pharmacogenomics, using an explicitly ancestry-informed framework. Under this approach, an Ancestry-Based Pharmacological Diagnosis (APD) could improve efficacy, safety, and equity, provided it is grounded in local evidence and clinically supported.

In this Perspective, APD refers to a clinically oriented framework that integrates individual- and population-level genetic ancestry information with actionable pharmacogenomics variants to inform drug selection, dosing, and risk stratification. APD does not constitute a deterministic algorithm or a race-based classification system; rather, it is an evidence-informed strategy that leverages ancestry as a modifier of pharmacokinetic and pharmacodynamic variability, with the goal of improving therapeutic efficacy, minimizing adverse drug reactions, and enhancing the clinical portability of pharmacogenomic guidance in genetically heterogeneous populations. Beyond ancestry-resolved allele frequencies, APD is intended to strengthen interpretation by incorporating haplotype/star-allele context and co-variant modifiers when available, acknowledging that the clinical penetrance of PGx variants can vary across genetic backgrounds. Crucially, prescribing recommendations remain genotype-based; ancestry is used to guide prioritization and calibrate evidence portability in admixed settings.

In the same way, “genetic ancestry” is used in in an explicitly operational sense that is widely adopted in population-genomic and admixture studies across Latin America and Colombia. In this literature (Rishishwar et al., 2015; Ruiz-Linares et al., 2014), ancestry is inferred by comparing individual genomic variation to reference source populations and summarizing ancestry as (i) continuous coordinates in principal component space and (ii) model-based admixture proportions representing contributions from major continental sources (commonly African, European, and Native American). This operationalization has been repeatedly used to describe the three-way admixture structure of Latin American populations and to quantify substantial heterogeneity in individual ancestry proportions, including in Colombian datasets (Mariño-Ramírez et al., 2025).

Accordingly, within APD, genetic ancestry is treated as a continuous and probabilistic genomic descriptor (e.g., PC-space position and admixture fractions, and when available, local ancestry at specific loci), rather than as ethnicity, geographic origin, or self-identified race (Bird et al., 2025). This distinction is central to our proposal: APD does not recommend therapy “because a person is Black/White/Indigenous,” but instead uses inferred ancestry patterns to contextualize population-structured allele frequencies and the likely portability of pharmacogenomic evidence across admixed subgroups.

## 6 CÓDIGO as a national enabler and proof-of-concept evidence

Colombia already has a key piece of infrastructure to operationalize an ancestry-based clinical pharmacogenomics approach: CÓDIGO (Consortium for Genomic Diversity, Ancestry, and Health in Colombia). This consortium integrates and harmonizes genomic data (WGS/WES/WGG) contributed by multiple institutions to strengthen local capacity in genomics, bioinformatics, and precision medicine, and disseminates results through a public platform (Mariño-Ramírez et al., 2025).

In its version 1.0, CÓDIGO aggregates 1,441 samples from 14 populations across 12 departments and enabled the characterization of >95 million variants, underscoring the clinical relevance of the country’s ancestral diversity. The resource provides summary statistics (allele frequencies and fractions of African/Indigenous/European ancestry) and even department-level frequencies for the 32 departments and Bogotá (the national capital), with query options by rsID (unique identifier for single nucleotide polymorphisms [SNP], gene, star allele haplotypes, or drug. This is directly useful for an ancestry-based diagnosis because it links regional ancestry heterogeneity with actionable pharmacogenomic variation, making it a foundational tool for research and analysis of these variants across different populations or in the context of specific diseases (Mariño-Ramírez et al., 2025).

This database has already been used in some studies to illustrate how genetic and ancestral heterogeneity can shape therapeutic efficacy and safety in pharmacological treatment. These analyses have identified significant differences in SNP frequencies between populations with different predominant ancestry (African vs. European) (Acosta-Monterrosa et al., 2025; Hernandez-Paez et al., 2025), with direct implications for drug selection and appropriate dosing.

*Case study 1*: Population-wide PharmGKB-CÓDIGO integration. Acosta-Monterrosa et al. (Acosta-Monterrosa et al., 2025) conducted a broad analysis integrating PharmGKB (a curated knowledgebase of genetic variants and their effects on drug response, including variant annotations and gene-drug pairs (Whirl‐Carrillo et al., 2021)) with CÓDIGO data. They identified ancestry-stratified differences in pharmacogenomics profiles and SNP allele frequencies between predominantly European-ancestry populations (e.g., Antioquia) and Afro-descendant populations (e.g., San Basilio de Palenque). Notably, the CYP3A4 variant rs3735451-C was present in 87.1% of individuals from Palenque but only 23.2% in the European-ancestry group, a pattern consistent with higher rivaroxaban exposure and a potentially increased bleeding risk in Afro-Colombian populations.

*Case Study 2*: Diabetes-associated SNPs in Colombian populations. Hernández-Páez et al. (Hernandez-Paez et al., 2025) assessed diabetes-relevant pharmacogenomics loci in Colombian populations and reported marked ancestry-associated differences in allele frequencies for variants linked to treatment response. For instance, rs7754840-C (associated with response to DPP-4 inhibitors such as sitagliptin) showed a frequency of 76.1% in the Afro-Colombian Palenque population compared with 28.4% in higher European-ancestry proportion groups, suggesting differential therapeutic response across ancestries. Similarly, rs8192675-C (associated with improved metformin response) was more frequent in the Afro-Colombian population (69.3% versus 35.9% in the higher European-ancestry proportion group).

These examples underscore the need to implement a pharmacological diagnosis grounded in genetic ancestry to optimize the treatment of conditions such as diabetes. San Basilio de Palenque, one of the populations analyzed in both studies, with its high proportion of African ancestry, cannot be directly extrapolated to Antioquia, a region with predominantly European ancestry. Recognizing this differentiation is essential to avoid dosing errors and to maximize therapeutic benefit, which would be critical for reducing risks and improving equity in access to personalized treatments.

## 7 Benefits and implementation value of APD in Colombia

The use of genetic ancestry in pharmacogenomics must be clearly distinguished from socially constructed notions of race. In APD, ancestry is treated as a continuous, probabilistic genomic attribute rather than a categorical or essentialist label. Its clinical use aims to reduce uncertainty in drug response prediction while avoiding genetic determinism or stigmatization. Importantly, APD is intended to complement, not replace, clinical judgment, environmental, and social determinants of health, and should be implemented within ethical frameworks that emphasize transparency, patient autonomy, data protection, and equitable access to precision medicine.

While several benefits outlined below are recognized advantages of pharmacogenomics broadly, APD contributes additional implementation value in Colombia by translating ancestry-resolved population heterogeneity into decisions about evidence portability, prioritization, and staged rollout across regions and subgroups. In this way, APD is framed not as a new justification for PGx, but as a practical approach to reduce miscalibration when PGx evidence is applied in a highly admixed setting, being compatible with pre-emptive PGx panels; its added value lies in ancestry-aware evidence calibration, highlighting where external findings may be miscalibrated, where local validation is most needed, and where implementation and pharmacovigilance efforts should be prioritized across heterogeneous Colombian subgroups.

**FIGURE 1:**
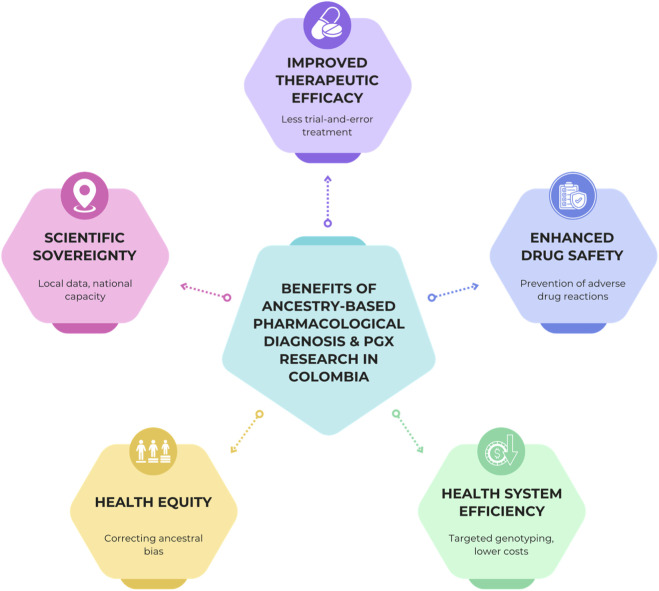
Benefits of expanding pharmacogenomic research and implementing ancestry-based pharmacological diagnosis in Colombia. Operationally, APD combines (i) pre-emptive/panel genotyping, (ii) model-based ancestry inference, and (iii) ancestry-aware evidence calibration to support evidence portability, prioritization, and staged rollout across regions and subgroups. The figure synthesizes the translational value of APD across five interconnected domains: 1) therapeutic efficacy, by reducing trial-and-error prescribing through ancestry-informed variant interpretation; 2) patient safety, via anticipation of ancestry-associated adverse drug reactions; 3) health-system efficiency, by prioritizing high-yield genotyping strategies aligned with population-specific allele frequencies; 4) health equity, by addressing historical underrepresentation of African- and Indigenous-ancestry populations in pharmacogenomics evidence; and 5) scientific sovereignty, by leveraging nationally led genomic resources such as CÓDIGO to generate locally relevant, implementation-ready knowledge. Together, these domains position APD as a scalable bridge between population genomics and routine clinical pharmacology. Source: authors.

In light of the considerations, we propose five potential country-level benefits of implementing an ancestry-based pharmacological diagnosis in clinical practice (Figure 1):

*1. Improved therapeutic efficacy:* Ancestry-related differences in efficacy variants can translate into distinct responses even under standard protocols. In diabetes, alleles associated with greater glycated hemoglobin reduction with DPP-4 inhibitors (e.g., CDKAL1 rs7754840-C) and with metformin (e.g., SLC2A2 rs8192675-C) are more frequent in the African-ancestry group than in the European-ancestry group (Hernandez-Paez et al., 2025). In practice, this enables more informed treatment selection and faster achievement of glycemic control by reducing repeated therapeutic trials. Within APD, these differences are used to calibrate evidence portability across Colombian subgroups and regions and to prioritize where local validation is most needed before routine implementation.
*2. Prevention of adverse events:* Knowledge of ancestry-specific frequencies of pharmacogenomics SNPs enables risk anticipation and more tailored therapeutic decisions. For example, the risk of thiazide-induced new-onset diabetes mellitus (NODM) could potentially be reduced, given that TCF7L2 rs7917983 has been associated with a higher likelihood of NODM among hypertensive patients treated with hydrochlorothiazide and shows an ancestry-differential pattern (more frequent in predominantly European than African ancestry groups). Similarly, in transplantation, tacrolimus use could be optimized because CYP3A5 rs776746 influences drug metabolism/exposure and has been associated with post-transplant diabetes, with substantially higher frequency in populations of predominantly African ancestry compared with those of predominantly European ancestry (Hernandez-Paez et al., 2025). In APD, such ancestry-differential patterns support risk calibration and targeted implementation in admixed contexts, reducing reliance on uniform extrapolation from external cohorts.
*3. Efficiency and cost savings for the health system:* Experimental evidence supports the clinical utility of pharmacogenomics panels: a European trial involving ∼7,000 patients reported that genotype-guided care reduced ADRs by 30% using a panel comprising 44 variants across 12 genes and 42 drugs (Pirmohamed, 2023). Accordingly, implementation in Colombia could be particularly impactful given the high costs associated with ADRs in internal medicine settings (Tribiño et al., 2006). Moreover, the performance and cost-effectiveness of pharmacogenomics implementation depend to a large extent on the allele frequencies of clinically relevant variants in the target population (Pirmohamed, 2023). APD translates ancestry-resolved heterogeneity into pragmatic rollout decisions, prioritizing where panel-based genotyping and downstream clinical workflows are likely to yield the greatest impact by region/subgroup rather than applying a uniform nationwide strategy. In Colombia, the ancestry-resolved mapping presented by Acosta-Monterrosa et al. (Acosta-Monterrosa et al., 2025) provides precisely the inputs needed to prioritize such targeting (summarizing frequencies, gaps, and potential clinical impact) and highlights scenarios in which findings from one population are not directly transferable to another, an evidence-portability problem that APD is explicitly designed to surface and mitigate.
*4. Health equity:* The global evidence base informing implementation remains skewed: 51.5% of participants (n = 336,073) across pharmacogenomics studies were European, whereas only 0.46% were reported as having African ancestry (n = 3,031) (Acosta-Monterrosa et al., 2025). Ignoring gaps in ancestral representativeness can perpetuate suboptimal clinical decisions and avoidable adverse events; therefore, integrating ancestry-stratified data is essential for practice. In Colombia, this entails expanding sampling toward populations with higher African and Indigenous ancestry, using prospective cohorts with longitudinal outcomes (response, plasma levels, ADRs) and replication/validation of actionable associations in high-risk groups, while aligning this agenda with explicit implementation and equity goals. APD strengthens this agenda by making evidence gaps and non-portability explicit at subgroup and regional levels, thereby prioritizing where recruitment, validation, and pharmacovigilance are most clinically consequential.
*5. Scientific sovereignty:* CÓDIGO strengthens local capacity and releases analysis-ready data, including allele frequencies and ancestry fractions, with annotations and query functionality by rsID, gene, drug, or haplotype (star alleles), even stratified at the departmental level. Given the region’s underrepresentation in genomics, it stands as a necessary country-level effort and reinforces national autonomy to design and validate an APD using locally generated evidence.

### 7.1 Limitations

APD is necessarily limited by the variants represented in available reference resources and clinical panels. As a result, it cannot anticipate private or extremely rare variants that are absent from current datasets and lack documented allele frequencies or clinical annotations. This underscores the importance of iterative updating using locally generated sequencing data and prospective outcome-linked cohorts to progressively improve coverage and clinical interpretability.

## 8 Implications for meta-research and scientific coherence in pharmacogenomics

Meta-research has emerged as a critical discipline focused on evaluating how scientific knowledge is produced, validated, synthesized, and translated, with particular attention to bias, reproducibility, transparency, and the alignment between research outputs and societal needs (Lozada-Martinez et al., 2025a; Lozada-Martinez et al., 2025b). A central insight from meta-research is that scientific systems can generate internally rigorous findings while still producing weak real-world value when evidence is fragmented, non-comparable, or poorly connected to downstream decisions (Lozada-Martinez et al., 2025c). These challenges intersect directly with evidence-based research, an approach that argues that new studies should be explicitly justified and designed in light of the totality of existing evidence, typically through systematic synthesis and structured evidence mapping, to reduce research waste, prevent redundant or misdirected studies, and increase the cumulative value of research (Robinson et al., 2021; Lozada-Martinez et al., 2024).

Within this combined framework, scientific coherence refers to the logical and methodological alignment across the full evidence pipeline: what questions are prioritized, which populations are sampled, how analyses are parameterized, how findings are synthesized, and how conclusions are translated into guidance (Lozada-Martinez et al., 2022). When pharmacogenomics evidence is generated predominantly in Eurocentric or genetically narrow samples and then extrapolated to highly admixed settings, incoherence arises between discovery, synthesis, and implementation (Lauschke and Ingelman-Sundberg, 2020). From a meta-research and evidence-based research standpoint, closing this coherence gap requires not only increasing representation, but also explicitly integrating population structure and ancestry-informed variability into study design, evidence synthesis, and guideline development, thereby improving comparability, portability, and cumulative knowledge building (Lozada-Martinez et al., 2024).

From a meta-research view, the proposal of an APD directly addresses a structural source of scientific incoherence in pharmacogenomics: the systematic misalignment between the populations in which evidence is generated and those in which it is clinically applied (Tafazoli et al., 2025; David et al., 2024). Eurocentric sampling biases, combined with inadequate consideration of ancestry-driven genomic heterogeneity, have produced pharmacogenomics knowledge that is internally valid but externally fragile when transferred to admixed or underrepresented populations (Tafazoli et al., 2025). APD reframes ancestry not as a descriptive variable, but as a coherence-enhancing modifier that improves the portability, reproducibility, and interpretability of pharmacogenomics evidence across heterogeneous clinical contexts (Shaaban and Ji, 2023).

From the standpoint of scientific coherence, APD provides a conceptual mechanism to reconcile discovery, validation, and implementation within a single translational continuum. By explicitly integrating ancestry at the population, regional, and individual levels, APD reduces the epistemic gap between variant-level associations and real-world therapeutic outcomes (Tafazoli et al., 2025; David et al., 2024; Shaaban and Ji, 2023; Russell et al., 2021). In doing so, it supports more coherent clinical decision-making, mitigates unjustified extrapolations, and strengthens the internal logic of precision pharmacology as an evidence-based discipline (Russell et al., 2021). More broadly, this approach illustrates how meta-scientific frameworks can guide the design of pharmacogenomics research agendas that are not only methodologically robust, but also context-aware, ethically grounded, and clinically consequential.

## 9 Conclusion

Taken together, Colombia’s pronounced regional and inter-individual ancestry heterogeneity argues for moving beyond one-size-fits-all pharmacogenomics toward an APD that is clinically actionable, ethically governed, and grounded in local evidence. Integrating ancestry information can improve therapeutic efficacy and safety and enhance the portability of genotyping panels and prescribing guidance, thereby reducing inappropriate extrapolations and suboptimal clinical decisions. Crucially, national resources such as CÓDIGO already provide the infrastructure to link ancestry patterns with actionable pharmacogenomics variants and to accelerate both discovery and implementation at scale. Harnessing this genomic diversity through rigorous research and responsible deployment, Colombia can turn ancestry-informed pharmacogenomics into a practical public-health tool, delivering safer, more effective therapies and a more equitable future for precision medicine.

## Data Availability

The original contributions presented in the study are included in the article/supplementary material, further inquiries can be directed to the corresponding author.
